# Attenuation Estimation and Acoustic Characterization of Mouse Lymph Node Tumor Using High-frequency Ultrasound

**DOI:** 10.1007/s11307-025-02007-2

**Published:** 2025-05-12

**Authors:** Masaaki Omura, Kazuki Maeda, Kazuki Tamura, Kenji Yoshida, Ariunbuyan Sukhbaatar, Tetsuya Kodama, Tadashi Yamaguchi

**Affiliations:** 1 Faculty of Engineering, University of Toyama, Toyama, Japan; 2 Graduate School of Biomedical Engineering, Tohoku University, Sendai, Japan; 3 Institute of Photonics Medicine, Hamamatsu University School of Medicine, Hamamatsu, Japan; 4 Center for Frontier Medical Engineering, Chiba University, Chiba, Japan

**Keywords:** High-frequency ultrasound, Attenuation coefficient, Acoustic characterization,, Mouse lymph node, Tumor growth

## Abstract

**Purpose:**

Lymph node (LN) biopsy is the gold standard for diagnosing metastasis. While ultrasound imaging is a non-invasive method for real-time LN metastasis diagnosis and tumor assessment, its accuracy depends on operator skill and system settings. Quantitative ultrasound can characterize tissue microstructure changes due to tumors, offering operator-independent parameters, and one of the quantitative ultrasound methods, the backscatter coefficient, is necessary to compensate for tissue attenuation. However, the change in the attenuation coefficient (AC) in the tumor growth is uncertain. Using in vivo high-frequency ultrasound (25 MHz) measurement and scanning acoustic microscopy (80 and 300 MHz) for ex vivo samples, we aim to investigate how tumor growth is linked to the attenuation and acoustic properties such as acoustic impedance and speed of sound related to ultrasonic wave propagation.

**Procedures:**

FM3 A-Luc mammary carcinoma cells were inoculated into the subiliac LNs of mice, and tumor progression was monitored over time. Bioluminescence imaging was used to assess tumor growth, while ultrasound measurements focused on estimating AC and other acoustic properties.

**Results:**

Results indicated that the mean of AC decreased, and its standard deviation increased as tumors grew, correlating with bioluminescence intensity. Furthermore, acoustic impedance and speed of sound varied between normal and tumor tissues, revealing differences in tissue microstructure from the histopathological images.

**Conclusions:**

The finding of a decrease in AC observed with tumor growth may play a crucial role in enhancing the accuracy of quantitative ultrasound on attenuation compensation, potentially improving the differentiation between metastatic and non-metastatic LNs.

## Introduction

The interaction of abnormal cells with malignant potential is essential for tumor development, subsequent progression, and the development of metastatic disease [1]. Assessing lymph node (LN) metastasis is a crucial aspect of diagnosing and treating cancer patients. LN biopsy is the current gold standard for diagnosing metastasis and determining its subtype.

Ultrasound imaging is a powerful modality for the non-invasive and real-time diagnosis of LN metastasis in examination and surgical situations. Some fundamental features based on the ultrasonic B-mode image such as the size, shape, and echogenicity of the LN have been differentiated between nonmetastatic and metastatic LNs [2, 3]. Doppler ultrasonography [4, 5], contrast-enhanced ultrasound imaging [6, 7], and elastography [8, 9] can accurately improve in differentiating between nonmetastatic and metastatic LN by focusing on centripetal or asynchronous perfusion, hyper- or heterogeneous enhancement, and perfusion defects [10], and elasticity of LN. However, independent operator skill and system setting are still required to achieve quantitative assessment. We hypothesize that quantitative ultrasound (QUS) considering the attenuation coefficient (AC) is beneficial in characterizing the change in tissue microstructure due to tumor growth.

QUS has been studied to assess tissue microstructure, e.g., fatty liver [11–13], skin lesion [14, 15], and hemorheology [16–18]. QUS algorithm is based on the ultrasonic backscatter signals derived from frequency spectral and envelope statistical features. The backscatter coefficient (BSC) that is computed from spectral estimation is related to scatterer size, concentration, shape, and acoustic impedance contrast. The BSC cancels system properties and compensated for attenuated property due to wave propagation. Hence, the BSC can characterize tissue microstructure with quantitative parameters that are independent of the operator skill or system setting.

High-frequency ultrasound has been useful in observing microstructures in superficial tissue such as skin [15, 19], vascular channel [20], and LN [21]. This is particularly beneficial for AC estimation, which is frequency-dependent and provides insight into tissue composition and microstructural alterations by high-frequency ultrasound. Previous studies have demonstrated that high-frequency AC imaging can enhance tissue differentiation [22]. Especially in previous studies for detecting lymph node metastases have been successful in ex vivo around 25 MHz ultrasound [22, 23]. In the development study, in vivo LN has been evaluated around 9 MHz ultrasound by QUS [24]. BSC-based parameters such as scatterer size and scatterer concentration are the most effective for accurately characterizing metastatic LN [24]. However, there is a limited assumption of the same AC as the typical value of soft tissues (0.5 dB/cm/MHz [25]) to compute BSC in evaluating both nonmetastatic and metastatic LN.

Ultrasonic attenuation refers to the gradual loss of energy that occurs as an ultrasonic wave propagation through a medium. This energy loss happens due to several factors, including scattering, absorption, and reflection of the wave propagation within the tissue. In biomedical ultrasound, attenuation is important because it affects how deeply the ultrasound can penetrate tissues and the clarity of the resulting images. Ultrasonic attenuation analysis is useful for breast tumor evaluation [26–28] and fatty liver disease diagnosis [29–31]. Until now, the AC of tumor tissue as well as its dependence on tumor growth in LN is not understood.

The acoustic properties, e.g., acoustic impedance and speed of sound (SoS), are related to the ultrasonic backscattered signals. Scanning acoustic microscopy (SAM) is effective in comprehending the relationship between acoustic properties and histopathological changes. Several studies using SAM have been introduced in biological tissues [32–34]. To predict microscopic histopathological structures, QUS parameters are compared to pathological changes in tissues [21, 35]. These findings have suggested that QUS parameter such as scatterer size is linked to the morphological change of cells in tumors [21]. Until now, the acoustic properties of tumor regions are unclear. It would be beneficial for the evaluation of tumor growth to understand the relationship between tissue microstructure and ultrasonic backscatter signals.

The motivation of this study is to demonstrate whether the AC changes with tumor growth of LN. Attenuation estimation was performed in the dataset with in vivo ultrasonic measurement using high-frequency ultrasound. The significance of the attenuation estimation is better feedback for attenuation compensation on QUS analysis. Secondly, the question about the relationship between acoustic properties and histopathological features of tumors is investigated in ex vivo LN using SAM. The basic acoustic properties such as acoustic impedance and SoS were compared with the histopathological image to predict the tissue microstructure with tumors in the LN.

## Materials and Methods

### Mouse and Celll

This study was approved by the Institutional Animal Care and Use Committee of Tohoku University and performed in accordance with published guidelines.

**Fig. 1.**
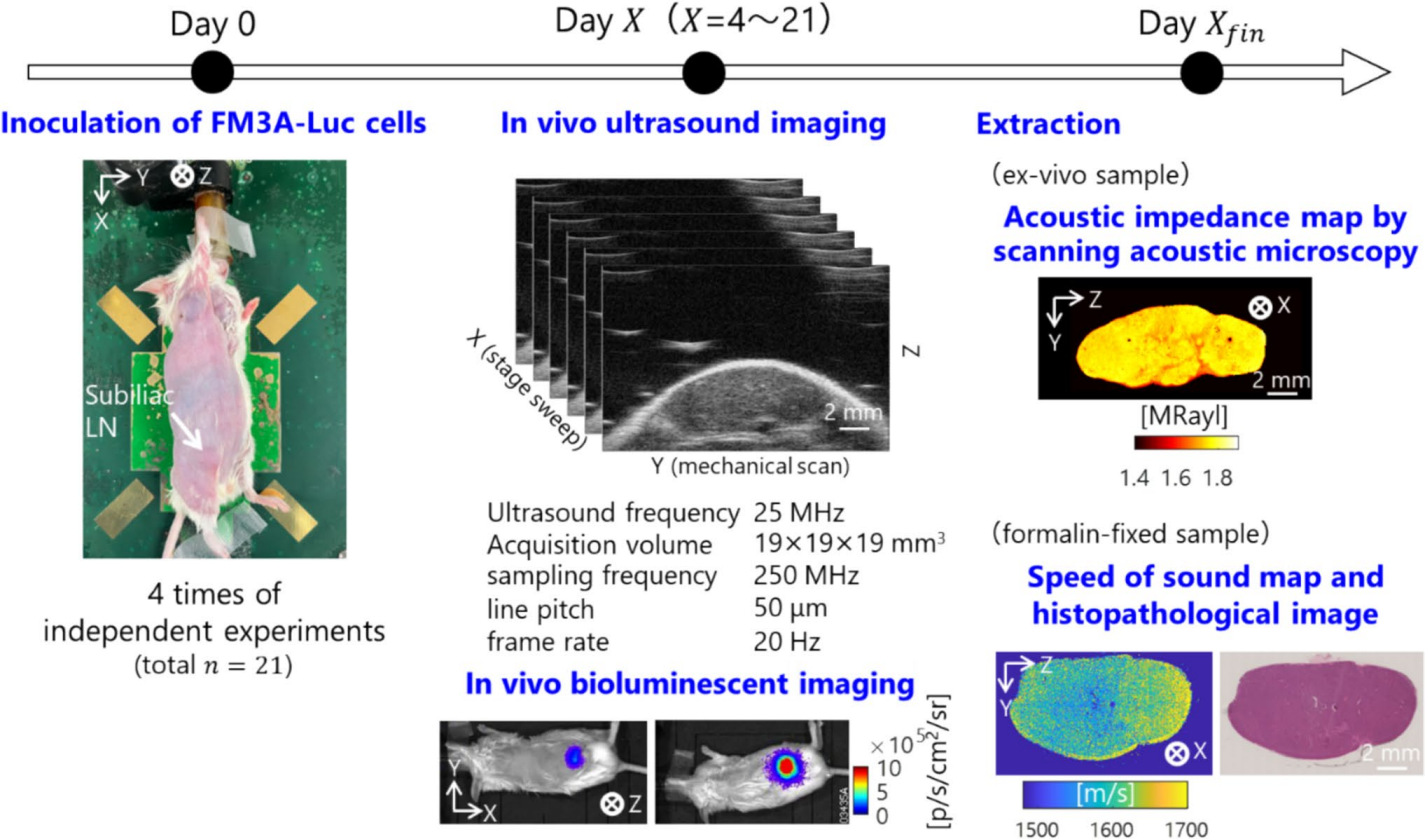
Schematic image of study design

**Fig. 2.**
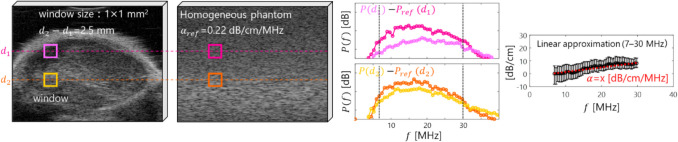
Schematic image of ultrasonic attenuation estimation

The overview of this study design is shown in Fig. 1. The subiliac LN (SiLN) of MXH10/Mo/lpr mouse was used as a pseudo-sentinel LN by inoculating it with a tumor. We used FM3 A-Luc cells, mouse mammary carcinoma cells, that constitutively express the luciferase gene as followed in our previous study [36]. The culture medium for FM3 A-Luc cells consisted of RPMI (Sigma-Aldrich) containing 10% fetal bovine serum, 1% L-Glutamine-P.S. Solution, and 0.5% Geneticin (G418, Sigma-Aldrich). The cell suspension was further diluted threefold with Matrigel, resulting in a final cell concentration of 3.3 × 10^5^ cells/mL. For the inoculation, mice were anesthetized with 2% isoflurane in air, and 60 µL of the cell suspension was directly bolus injected into the SiLN by a 27G Nipro Myjector syringe. (Figure 2)

### In Vivo Measurement

Ultrasonic measurement and bioluminescence imaging were conducted on the following day in each experiment: Group I ($n$= 4) on days 0, 7, 14, and 21 with the day of tumor cell inoculation designated as day 0. For the reproducibility of this study, the additional data collection was performed on Group II ($n$ = 5) on days 0, 4, 6, 8, and 11; and Group III ($n$= 5) on days 0, 4, 7, 10, 13, 16, and 20; Group IV ($n$ = 7) on days 0, 7, 10, 13, and 16.

To evaluate tumor growth, we used a bioluminescence imaging system (IVIS Lumina LT series III, Perkin Elmer). Mice were anesthetized with 2% isoflurane, and 15 mg/mL luciferin (D-Luciferin Potassium Salt, FUJIFILM Wako) was injected intraperitoneally. Bioluminescence intensity was measured 10 min later. The average luminescence [10 log_10_(intensity)] per unit time was calculated using the accompanying analysis software (Living Image, Perkin Elmer).

The mouse was also anesthetized and positioned on a heated stage for in vivo ultrasound imaging. The ultrasound scanner (Vevo 770, FujiFilm) with a mechanical single-element transducer (RMV- 710B, VisualSonics, FujiFilm) was used to observe LN and collect A/D converted data. The center frequency of the single-element transducer is 25 MHz. The pitch of scanline imaging is 0.05 mm. A laboratory-made data acquisition system for radio-frequency (RF) signals was constructed using a Vevo 770 and an A/D board (Sbench 6, Spectrum) with a sampling frequency of 250 MHz and 14-bit digitization. A single-element transducer was mechanically scanned across Y-axis of 19 mm (380 pixels) and X-axis of 19 mm (190 pixels) using a motorized stage synchronized with the A/D board. The frame rate was 20 fps. The acquired time for data curation was around 10 s. Three-dimensional reconstructed RF data was used to estimate the local AC.

### Ex Vivo Measurement

After a sacrifice on the final day of in vivo measurement, the SiLN was extracted for acoustic characterization. The cross-section at the maximum surface of the LN was divided as raw (for acoustic impedance) and formalin fix samples (for SAM and histopathology).

For the comprehension of acoustic impedance in microscopic structure, RF data (8 bits and 2 GS/s sampling frequency) were measured from ex vivo tissue cross-sections via distilled water on a polystyrene dish by a 80 MHz single-element transducer (HD80–1.2–2.5, Toray). The data acquisition system was modified SAM (modified AMS- 50 SI, Honda Electronics). There is a limitation that the scanning area was 4.8 mm × 4.8 mm (scanning pitch of 16 μm) at the maximum. Hence, the scanning area was readjusted to two dimensions (2D) to observe the whole spatial distribution in the cross-sections of the LN. All acquisition protocols were followed by the previous study [37] using commercial software (UMScope, Honda Electronics). The acoustic impedance was analyzed using the reference medium theory [38].

After formalin fixation of the ex vivo LN, the samples were embedded in paraffin and sectioned into continuous 7 μm thick slices for SoS analysis and pathological observation. The dataset for SoS analysis was acquired using a laboratory-made scanner [39] with a 300 MHz single-element transducer (HT- 400 C, Honda Electronics). Briefly, RF signals from a sliced specimen via distilled water on a glass plate were obtained through 2D scanning of the entire lymph node region, with a sampling frequency of 2.5 GHz and 12-bit digitization. The SoS was calculated by the time-of-flight of RF signals separated by an autoregressive model [40]. The pathological specimens were stained using the hematoxylin–eosin method and observed with a virtual slide scanner (NanoZoomer S60, Hamamatsu Photonics) equipped with a 40 × magnification lens.

### Attenuation Estimation

The local AC was computed using the log-difference method using reference phantom [41, 42]. The local window with the size of 1.0 mm^2^ [336 (in Z-axis) × 20 (in Y-axis)] pixels] was scanned within the internal LN. To consider the motion artifact due to cardiac beat, the scanning frame in the presence of displacement was eliminated to estimate the local AC. The LN region was initially grouped by superpixels [43] and segmented by k-means method [44] into background, skin, and internal LN.

The power spectrum $P$ of the backscattered signal can be defined by the system's transmission-reception characteristics $S$, $BSC$, and the attenuation characteristic of the exponential term as $P\left(f\right)=S\left(f\right)\bullet BSC\left(f\right)\bullet {e}^{-4\alpha df}$. When taking the natural logarithm of the equation, it can be defined as $\text{ln}\left[P\left(f\right)\right]=S\left(f\right)+BSC\left(f\right)-4\alpha df$. Considering the frequency characteristics of the backscattered waves in the LN, ${P}_{s}$ relative to the reference one ${P}_{ref}$ at arbitrary depths ${d}_{1}$ and ${d}_{2}$,

1 $$\left.\text{ln}\left(\frac{{P}_{s}\left(d,f\right)}{{P}_{ref}\left(d,f\right)}\right)\right|{d}_{1}=4{(\alpha }_{ref}-{\alpha }_{p}){d}_{1}f+\Delta BSC(f)$$

2 $$\left.\text{ln}\left(\frac{{P}_{s}\left(d,f\right)}{{P}_{ref}\left(d,f\right)}\right)\right|{d}_{2}=4{(\alpha }_{ref}-{\alpha }_{p}){d}_{2}f+\Delta BSC(f)$$

Here, assuming that the BSC is constant at ${d}_{1}$ and ${d}_{2}$, solving from Eqs. (1) and (2), we obtain

3 $${\alpha }_{s}f=8.686\frac{\left.\text{ln}\left(\frac{{P}_{s}\left(d,f\right)}{{P}_{ref}\left(d,f\right)}\right)\right|{d}_{1}-\left.\text{ln}\left(\frac{{P}_{s}\left(d,f\right)}{{P}_{ref}\left(d,f\right)}\right)\right|{d}_{2}}{4\left({d}_{2}-{d}_{1}\right)}+{\alpha }_{ref}f$$

The equation allows for the calculation of the frequency dependence of the attenuation as described below. Furthermore, by performing a linear approximation of the attenuation obtained from Eq. (3), the slope can be derived as the AC ${\alpha }_{s}$. The useful frequency range was 6–31 MHz at $-$ 20 dB bandwidth. The AC ${\alpha }_{ref}$ of reference phantom was 0.22 dB/cm/MHz in the total attenuation estimation using the reflector method [45]. The distance between ${d}_{1}$ and ${d}_{2}$ was 2.5 mm to include the whole region of the LN.

### Acoustic Characterization with Acoustic Impedance and Speed of Sound

Secondly, for the comprehension of the acoustic property and histopathology, we spatially compared acoustic impedance, SoS, and pathological images. The acoustic impedance ${Z}_{s}$ was calculated from both the reference RF signal (negative-peak voltages ${A}_{ref}$) with known acoustic impedance (${Z}_{0}$ and ${Z}_{ref}$), and the sample signal ${A}_{s}$ from the cross-sectional sample [38] as follows

4 $${Z}_{s}=\frac{\left(1-\frac{{A}_{s}}{{A}_{ref}}\frac{{Z}_{ref}-{Z}_{0}}{{Z}_{ref}+{Z}_{0}}\right)}{\left(1+\frac{{A}_{s}}{{A}_{ref}}\frac{{Z}_{ref}-{Z}_{0}}{{Z}_{ref}+{Z}_{0}}\right)}{Z}_{ref}$$

The acoustic impedance of the purified water, ${Z}_{0}$, and that of the polystyrene dish, ${Z}_{ref}$, were set at 1.50 and 2.35 MRayl, respectively [38].

The RF data from the specimen for SoS analysis was firstly separated with two signals: distilled water – surface of the specimen (first wave) and bottom of specimen – glass plate (second wave) using a fifth-order autoregressive mode model [39]. The time-of-flight between specimen and reference (i.e., distilled water – glass plate signals without specimen) data was calculated by

5 $${c}_{s}=\frac{\Delta {t}_{1}}{\Delta {t}_{2}-\Delta {t}_{1}}{c}_{ref},$$

where $\Delta {t}_{1}$ and $\Delta {t}_{2}$ represent the time-of-flight between the first and reference waves, and the second and reference waves, respectively.

## Results

**Fig. 3.**
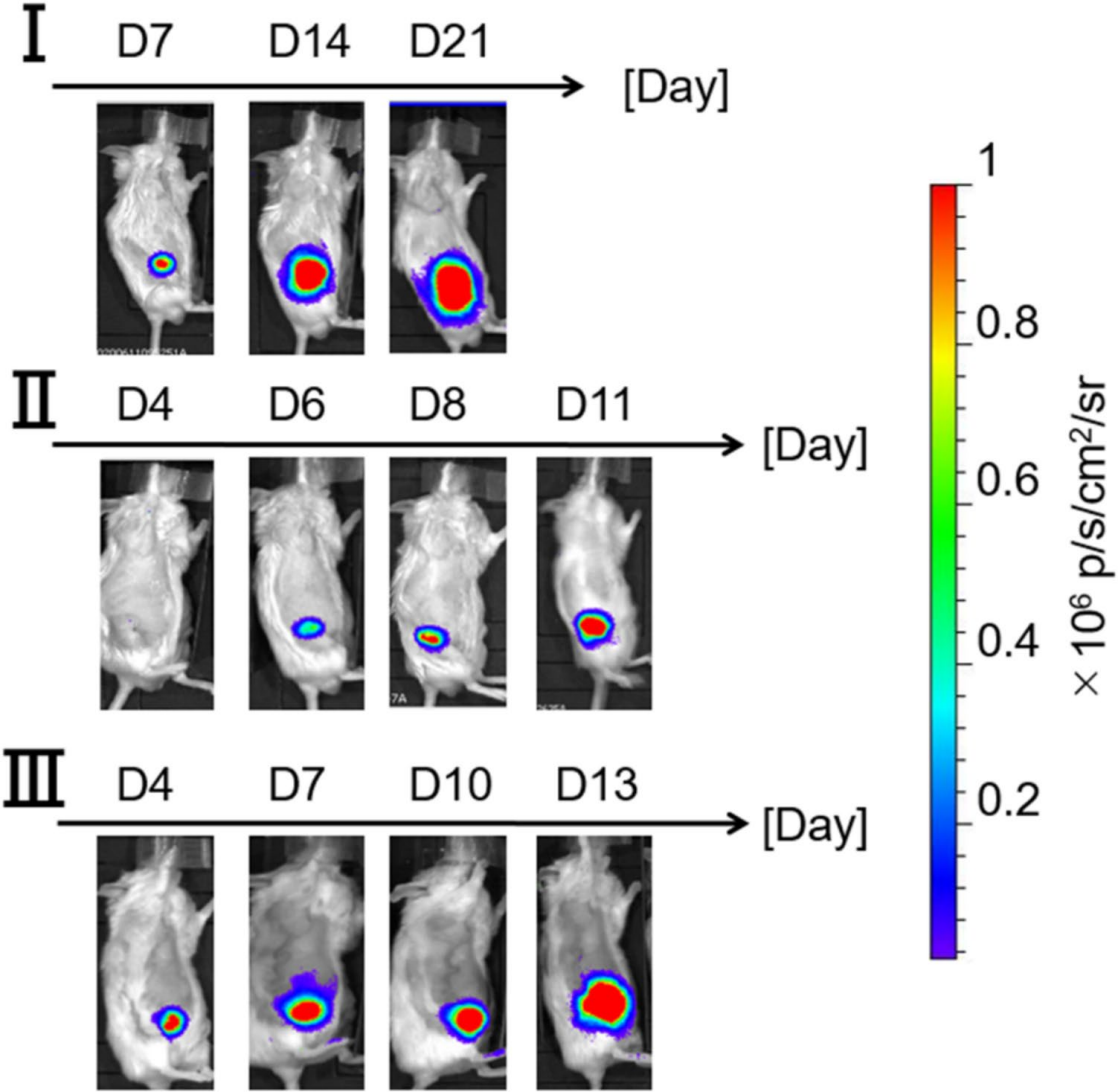
Spatial distribution of intensity in bioluminescence imaging (groups I, II, and III)

**Fig. 4.**
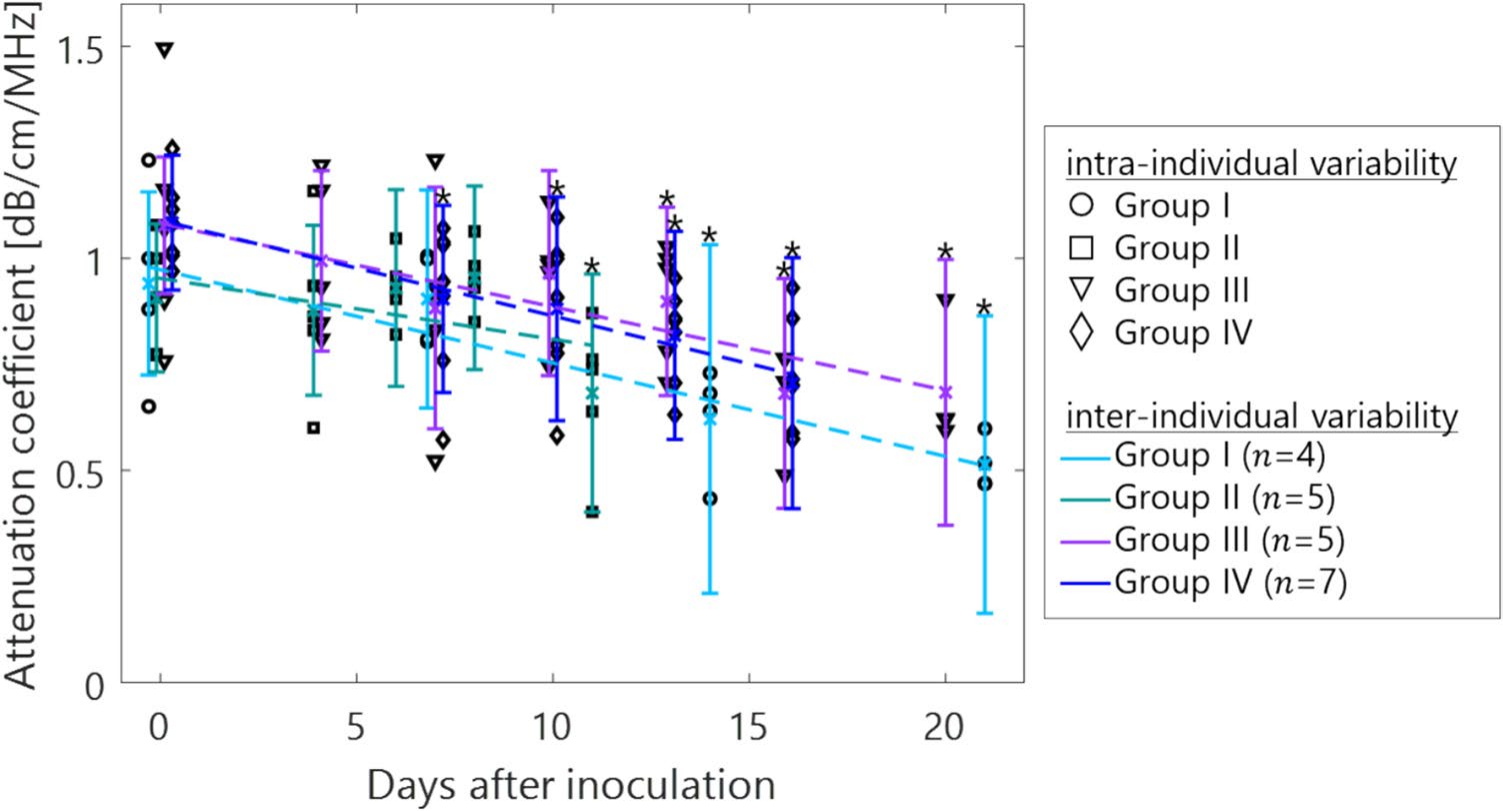
AC estimates in days after inoculation of tumor cells. Each marker shows the mean of the local AC in each mouse LN. The linear regression was performed using the mean of the local AC as shown in the dashed line (coefficient of determination = 0.61). The marker shows the mean of the local AC within four groups of experiment. The SD of the local AC was abbreviated for clarity, but the error bar indicates the mean of the SD of the local AC within four groups of experiment. One-way ANOVA and Tukey’s post-hoc test were examined in each experiment; *$p$ < 0.05, day 0 vs. days 14 and 21 in Group I; day 0 vs. day 11 in Group II; day 0 vs. days 13, 16, and 20 in Group III; day 0 vs. days 13 and 16 in Group IV.

**Fig. 5.**
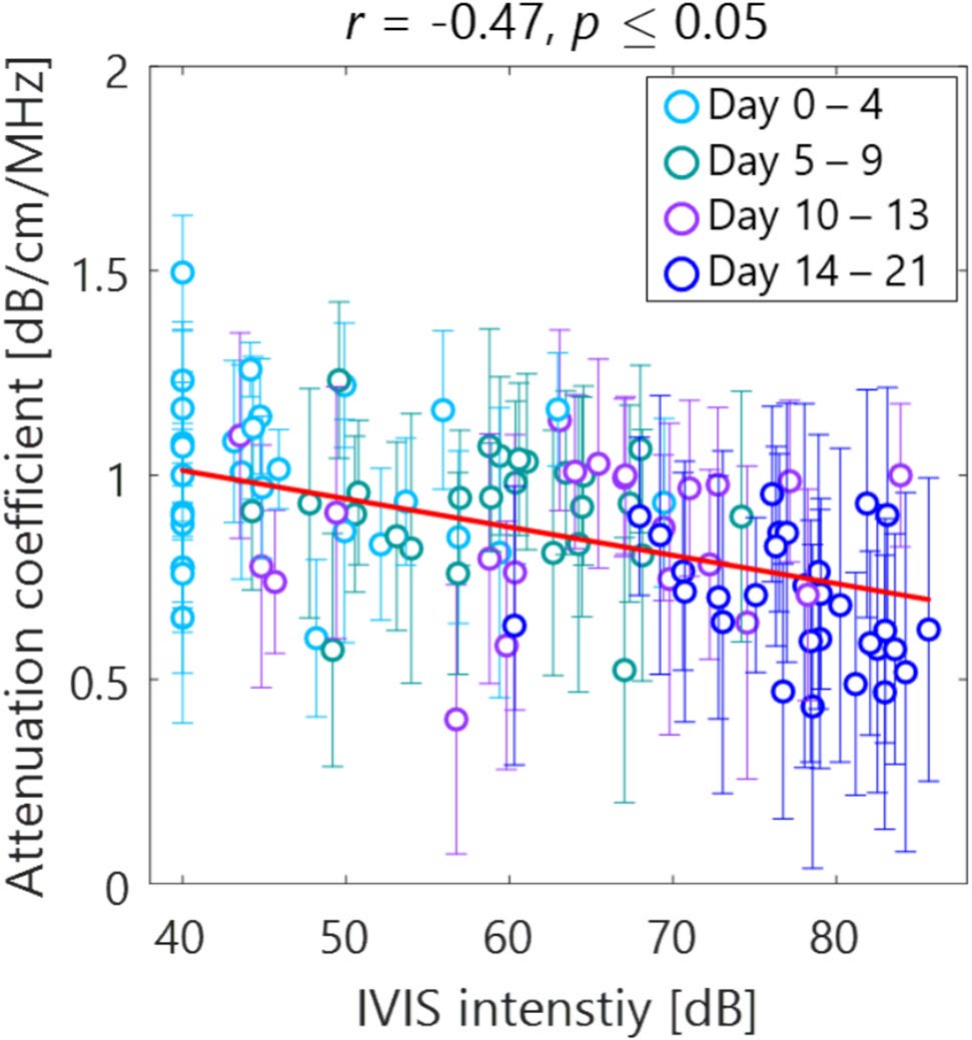
Scatter plots of the AC estimates and bioluminescence intensity in each mouse LN. Each plot and error bar shows mean and SD of spatial variability within individual data. Tukey's multiple comparison test was used, and Wiseman's rank correlation coefficient $r$ was used for the correlation between AC and luminescence intensity (red line).

Figure 3 displays the example of bioluminescence intensity map in days after inoculation. The intensity in the LN increased with days after inoculation in each group of experiments. Figure 4 shows the AC estimates for each mouse LN, and between LNs for each experiment. Overall, the AC decreases with days after inoculation, with a maximum decrease from 1.0 to 0.6 dB/cm/MHz. Also, the standard deviation (SD) of the AC estimates increased with days after inoculation, with a maximum increase from 0.22 to 0.35 dB/cm/MHz. Figure 5 shows the scatter plots of AC estimates and bioluminescence intensity in each mouse LN. The bioluminescence intensity was higher over time after inoculation corresponding to the spatial distribution in Fig. 3. The AC also decreases with increasing bioluminescence intensity ($r=-0.47$, $p\le$ 0.05). In addition, both the luminescence intensity and AC are scattered in the early stage of tumor growth in light blue, green, and purple, while they show a stable trend after blue day 14, which is assumed to indicate a strong tendency toward tumorigenesis.

**Fig. 6.**
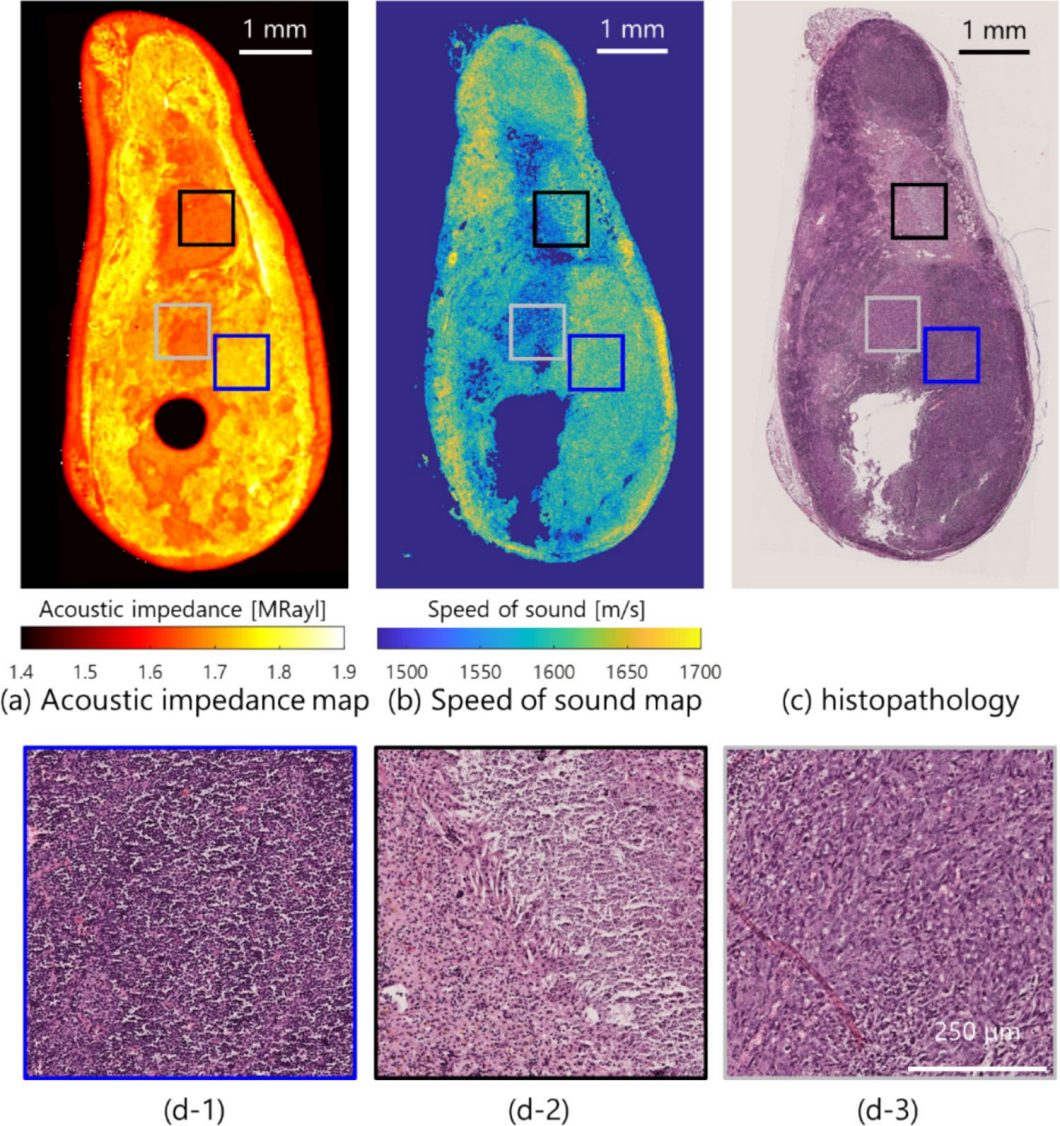
Example of (**a**) acoustic impedance, (**b**) speed of sound, and (**c**) histopathological images in ex vivo LN on day 21 of group I. The spatial images were aligned by the image registration using the function “registrationEstimator” on MATLAB. Enlarged histopathological images in typical three regions of (**c**): (**d-1**) Parenchyma tissue, (**d-3**) tumor tissue with fibrosis, (**d-2**) tumor tissue with meristem

Figure 6 displays the example of acoustic impedance, SoS, and histopathological images in the two-dimensional cross-section. The structures of cells differed between parenchyma and tumor tissues, with dense cell number density in the parenchymal tissue, whereas sparse cell number density was observed in the tumor tissue of the histopathological image as shown in Figs. 6(d- 1)–(d- 3). Especially in Fig. 6(d- 2), the inhomogeneous distribution with tumor and fibrotic tissues was observed. In these regions, the acoustic properties were different between parenchyma and tumor tissues (minimum–maximum values); 1.75–1.85 MRayl, 1600–1650 m/s in parenchyma tissue in Fig. 6(d- 1); 1.60–1.80 MRayl, 1500–1650 m/s in tumor tissue with fibrosis in Fig. 6(d- 2); 1.60–1.70 MRayl, 1500–1550 m/s in tumor tissue in Fig. 6(d- 3).

## Discussion

In this study, we compared tumor growth by bioluminescence intensity to the AC estimates by ultrasonography. Bioluminescence intensity in the LN increased with time, which shows the tumor growth as shown in Figs. 3 and 5. Hence, the AC overall decreased with tumor growth in each experimental group.

The impact of the differences in AC obtained on the BSC analysis would contribute to differentiating with tissue properties such as tumor growth and metastasis. Because a decrement in the AC results in a smaller slope of the attenuation correction term, the spectral slope on the BSC (e.g., Fig. 4(b) in [22]) also decreased in the metastatic LN. It is known that this difference in the spectral slope or scatterer size is the most important feature contributing to the discrimination between non-metastatic and metastatic LN [24]. However, if tumor formation causes the AC to decrease against healthy LN, it is expected that the spectral would decrease, which might enhance the discrimination capability between metastatic and non-metastatic LN.

Acoustic characterization with acoustic impedance and SoS was preliminarily investigated to presume the tissue microstructure in local tumor regions. We hypothesized that the main components of the tissue microstructure were parenchyma tissue, tumor tissue with fibrosis, and tumor tissue with meristem as shown in Fig. 6. In these tissues, the acoustic impedance and SoS were locally different, which showed the sparse tissue microstructure of degeneration in the tumor tissue. Acoustic impedance is the product of density and SoS. A reduction in either or both factors occurs in tissues with a higher content of fat or fluid. Moreover, the SoS tends to be lower in tissues that are less dense or have a higher fat or fluid content. For example**,** fatty liver or edematous (fluid-swollen) tissues tend to have lower SoS and lower acoustic impedance compared to healthy tissue [33, 39, 46]. We think that the difference in microscopic acoustic properties between parenchyma and tumor tissues is distinct from the features of the AC in the whole region of the LN. Although the validation of several samples was limited, the acoustic characterization preliminary could reveal the difference between normal and tumor tissues.

The selection of imaging frequencies was based on the trade-offs between resolution, penetration depth, and attenuation. For in vivo imaging, 25 MHz provides an optimal balance between penetration depth of around 10 mm and spatial resolution around 0.05 mm. At this frequency, ultrasound could allow for full lymph node visualization while maintaining the resolution. Also, previous study succeeds in detecting lymph node metastasis using 26 MHz ultrasound [23]. For ex vivo acoustic microscopy, 80 MHz and 300 MHz were chosen to achieve high-resolution characterization of tumor microstructure with stable measurement. At 80 MHz, the resolution improves to approximately 20 µm, allowing differentiation of small tissue features while maintaining sufficient reflected signals from bulk tissue sections. In our previous study, the acoustic impedance was successfully obtained at 80 MHz [37, 46]. At 300 MHz, the resolution further increases to the subcellular level around 5 µm, enabling detailed assessment of acoustic heterogeneity and cellular morphology corresponding to the histopathology [39, 47].

Naturally, a tumor is an abnormal growth of cells, and metastasis is the process by which malignant tumors spread from their original site to other parts of the body. There is a limitation that the metastatic LN such as proper axillary LN in our mice model [48] was not characterized in this study. Because metastatic LN in our mice model has more local distribution of metastasis, the attenuation estimation would suffer from the severe tradeoff between spatial resolution and precision of the AC estimates [49, 50]. In this study, the frequency spectral analysis was performed in three dimensions to evaluate the AC estimates in the whole region of the LN. In contrast, the acoustic characterization and histopathology were only observed in two dimensions of the cross-section at the maximum surface due to the fundamental principle of the SAM. Image registration is a challenge to compare the spatial distributions between the AC and microscopic acoustic characterization in several sliced samples.

The log-difference method assumes relatively uniform tissue properties such as BSC, which may not fully account for the heterogeneity present in tumor-infiltrated lymph nodes. However, we selected the local window (1.0 mm^2^) within the lymph nodes, minimizing the influence of heterogeneous acoustic properties. The size of the local window was considered in 17 $\lambda$ × 17 $\lambda$ (wavelength $\lambda$ at 1540 m/s) to ensure the robustness of AC estimation [50]. The spatial feature of tumor infiltration within the size of 1.0 mm^2^ could be observed in the typical image of acoustic characterization and histopathology as shown in Fig. 6. Although the typical region of the tumor could not be confirmed in the local AC, the SD of the AC estimates increased in Fig. 4. To further address this concern, future work could incorporate alternative techniques such as spectral-based attenuation estimation [51] and model-free approach for heterogeneous tissue [52].

In this study, we performed in vivo measurements using mechanical scanning due to limitations of data acquisition system. Regarding the target displacement, we eliminated the scanning frame in breathing variation to estimate the AC. The continuous measurement and analysis in 3D were limited in this mechanical scanning around 20 fps. Ultrasound systems have been widely accelerated by matrix and row-column array probes for ultrafast imaging around 1000 fps under 10 MHz [53, 54], and the current high-frequency linear array probe over 20 MHz [55] is also useful to scan 2D image for ultrafast imaging in future works.

## Conclusions

This study investigated the potential of using quantitative acoustic properties, such as AC and acoustic impedance, to assess tumor growth and microstructural changes in the LN. Our findings demonstrated that the AC decreases as tumor growth progresses, correlating with bioluminescence intensity, which serves as tumor development. The spatial distribution of acoustic properties, specifically acoustic impedance and SoS, varied between healthy and tumor tissues, indicating distinct tissue microstructures. Moreover, the decrease in AC observed with tumor growth may play a crucial role in enhancing the accuracy of QUS analysis, potentially improving the differentiation between metastatic and non-metastatic LNs. Further studies are needed to validate these findings in metastatic LNs and to explore the three-dimensional acoustic properties.

## Data Availability

All research data and computer codes are available from the corresponding author upon request.
